# Highly sensitive electrochemical immunosensor based on electrodeposited platinum nanostructures confined in silica nanochannels for the detection of the carcinoembryonic antigen

**DOI:** 10.3389/fchem.2023.1271556

**Published:** 2023-10-20

**Authors:** Qinping Guo, Xue Fan, Fei Yan, Yinquan Wang

**Affiliations:** ^1^ Shanxi Bethune Hospital, Shanxi Academy of Medical Sciences, Tongji Shanxi Hospital, Third Hospital of Shanxi Medical University, Taiyuan, China; ^2^ Key Laboratory of Surface and Interface Science of Polymer Materials of Zhejiang Province, Department of Chemistry, Zhejiang Sci-Tech University, Hangzhou, China

**Keywords:** platinum nanoparticles, silica nanochannel films, electrochemical immunosensor, carcinoembryonic antigen, anti-fouling detection

## Abstract

In this study, we report a highly sensitive electrochemical immunosensor for carcinoembryonic antigen (CEA) detection based on the electrodeposited platinum nanoparticles (Pt NPs) confined in the ultrasmall nanochannels of vertically ordered mesoporous silica film (VMSF). VMSF bearing amine groups (NH_2_-VMSF) can be prepared on the indium tin oxide electrode surface via a one-step co-condensation route using an electrochemically assisted self-assembly method, which renders a strong electrostatic effect for [PtCl_6_]^2-^ and leads to the spatial confinement of Pt NPs inside the silica nanochannels after electrodeposition. The external surface of NH_2_-VMSF is functionalized with CEA antibodies using glutaraldehyde as a coupling agent, resulting in an electrochemical immunosensing interface with good specificity for CEA detection. Under optimal experimental conditions, high affinity between the CEA antibody and CEA produces a steric hindrance effect for the accessibility of the electrochemical probe ([Fe(CN)_6_]^3-^) in the bulk solution to the underlying indium tin oxide surface, eventually resulting in the attenuated electrochemical signal and enabling the detection of the CEA with a wide linear range of 0.01 pg/mL∼10 ng/mL and a pretty low limit of detection of 0.30 fg/mL. Owing to the signal amplification ability of Pt NPs and the anti-biofouling property of NH_2_-VMSF, the as-prepared electrochemical immunosensor based on the Pt NPs@NH_2_-VMSF displays an accurate analysis of the CEA in human serum samples, holding significant promise for health monitoring and clinical diagnosis.

## 1 Introduction

Nanomaterials usually exhibit a large specific surface area and unique magnetic/optical/electrochemical properties that can improve the sensitivity and selectivity of various chemo/biosensors (Mao et al., 2019; Xi et al., 2019; Qiu et al., 2021; Zheng et al., 2021; Huang Y. et al., 2023; Xu et al., 2023; Zhu et al., 2023). Among them, noble metal nanoparticles (NPs) with nanosized structures have garnered significant attention due to their unique physicochemical properties and large specific surface area compared to their bulk materials, which have been widely used in electroanalytical applications (Lin et al., 2020; Zhao et al., 2020). Platinum (Pt) NPs are a kind of commonly used noble metal NP but unstable and will aggregate because of their high surface energy, resulting in the disappearance of specific desired features at the ultrasmall nanoscale. To control Pt NPs at the nanoscale, organic ligands or particular supported materials are introduced in the preparation procedure (White et al., 2009).

Recently, nanoporous materials have gained significant attention due to their high specific surface area, adjustable structure, and pore size and have exhibited considerable potential in applications such as adsorption/separation, sensing, catalysis, and energy storage applications (Cui et al., 2020; 2021; Zhao et al., 2020; Gong et al., 2022a; Liu et al., 2022). Vertically ordered mesoporous silica films (VMSFs) are a kind of solid nanoporous materials composed of highly ordered and uniform silica nanochannels (2–3 nm in diameter and tens to a hundred nanometers in length) and high porosity (Walcarius, 2021; Huang J. et al., 2023; Deng et al., 2023). In the past decades, an increasing number of electrochemical and electrochemiluminescence sensors have been designed using VMSF as the electrode-modified material (Liang et al., 2021; Su et al., 2022; Wei et al., 2022; Zhou et al., 2022). Although VMSF has unique insulating properties, it has been extensively utilized as an electrode protective layer for the direct and highly sensitive anti-biofouling analysis of complicated media (Wang et al., 2022; Zheng et al., 2022; Zhu et al., 2022). On the one hand, VMSFs bearing a large amount of silanol groups (p*K*_a_ = 2–3) display pronounced permselective effects toward targets or probes and simultaneously have excellent molecular sieving capability for them (Luo et al., 2022; Lv et al., 2022). On the other hand, they offer a lot of tiny confined spaces for the synthesis of metal NPs (e.g., gold (Ding et al., 2014a; Huang L. et al., 2023), Pt (Ding and Su, 2015; Li et al., 2020), and nickel (Ding et al., 2020)), polymers (Ding et al., 2014b), and graphene quantum dots (Zhang C. et al., 2023). The Su group employed two methods (namely, direct electrodeposition and chemical reduction) to achieve Pt NPs in silica nanochannels (Ding and Su, 2015; Li et al., 2020). The latter one needs first confinement of polyaniline polymer inside the nanochannels, generating the secondary and tertiary imines for easy complex with PtCl_6_^2–^ and subsequently suffering from chemical reduction *in situ*. To the best of our knowledge, simple modification of VMSF with functional groups for adequate incorporation of PtCl_6_^2–^ and further electrodeposited growth of Pt NPs has not yet been reported. Moreover, VMSF with functional groups renders the binding site for immobilization of specific recognition elements, exhibiting promising ability for the development of various sensitive and selective electrochemical sensors (Gong et al., 2022b; Ma et al., 2022b; Zhang T. et al., 2023; Chen et al., 2023).

Screening of tumor markers in human serum, especially the level of the carcinoembryonic antigen (CEA), is particularly valuable for the early auxiliary diagnosis and prognosis of various cancers (Zhang et al., 2022; Zhou et al., 2023). CEA concentration in the blood serum of healthy individuals is generally below 5 ng/mL, whereas cancer patients may exhibit levels exceeding 20 ng/mL (Lin et al., 2021). Several strategies have been developed for CEA detection, such as the enzyme-linked immunosorbent assay (Song et al., 2017), electrochemiluminescence method (Zhang et al., 2017), immunohistochemical method (Zeng et al., 1993), radioimmunoassay (Edgington et al., 1976), fluoroimmunoassay (Huang et al., 2018), and electrochemical immunoassay (Tang et al., 2007; Yan et al., 2023). Among them, electrochemical immunoassay has many advantages because of its low cost, rapid response, high selectivity, easy operation, and portability. Therefore, the development of electrochemical sensors with high sensitivity and anti-fouling capacity for direct detection of the CEA in human serum is highly desirable.

In this work, we demonstrate the use of amino group-functionalized VMSF (NH_2_-VMSF) for the confined synthesis of Pt NPs and the design of a highly sensitive electrochemical immunosensor for the CEA. NH_2_-VMSF-carrying amino groups provide a strong electrostatic effect for [PtCl_6_]^2−^ and lead to the well confinement of Pt NPs after electrodeposition. Such obtained Pt NPs confined in ultrasmall nanochannels of NH_2_-VMSF (termed as Pt NPs@NH_2_-VMSF) can be obtained in several seconds using a simple and controllable electrochemical method. The external surface of NH_2_-VMSF is immobilized with CEA antibodies using glutaraldehyde as a coupling agent, giving rise to an electrochemical immunosensing interface with good specificity for CEA detection. Due to the high affinity between the CEA antibody and CEA on the sensing interface, the steric hindrance effect is enhanced for the accessibility of the electrochemical probe ([Fe(CN)_6_]^3−^) in the bulk solution to the underlying ITO surface, ultimately yielding the relationship between the attenuated electrochemical signal and CEA concentration. Benefiting from the signal amplification ability of Pt NPs and the anti-biofouling property of NH_2_-VMSF, the developed electrochemical immunosensor based on Pt NPs@NH_2_-VMSF can be applied to sensitively and selectively detect CEA in human serum.

## 2 Materials and methods

### 2.1 Chemicals and materials

The CEA antigen and anti-CEA antibody, prostate-specific antigen (PSA), and alpha-fetoprotein (AFP) were purchased from Beijing Key-Bio Biotech Co., Ltd. (Beijing, China). S100 calcium-binding protein *β* was bought from Proteintech (Wuhan, China). C-reactive protein (CRP) was ordered from Nanjing Okay Biotechnology Co., Ltd. (Nanjing, China). Hexadecyl trimethyl ammonium bromide (CTAB), silicon tetraacetate (TEOS), potassium ferricyanide (K_3_[Fe(CN)_6_], 99.5%), potassium ferrocyanide (K_4_[Fe(CN)_6_], 99.5%), sodium dihydrogen phosphate dihydrate (NaH_2_PO_4_·2H_2_O), sodium phosphate dibasic dodecahydrate (Na_2_HPO_4_·12H_2_O), glutaraldehyde (GA), and chloroplatinic acid hexahydrate (H_2_PtCl_6_·6H_2_O) were received from Aladdin Biochemical Technology Co., Ltd. (Shanghai, China). 3-Aminopropyltriethoxysilane (APTES) and potassium hydrogen phthalate (KHP) were purchased from Shanghai Macklin Biochemical Co., Ltd. (Shanghai, China). Sodium nitrate (NaNO_3_), sodium hydroxide (NaOH), and ethanol (99.8%) were purchased from Hangzhou Gaojing Fine Chemical Co., Ltd. (Hangzhou, China). Concentrated hydrochloric acid (HCl) and concentrated sulfuric acid (H_2_SO_4_) were obtained from Shuanglin Inorganic Chemical Plant (Hangzhou, China). Phosphate buffer solution (PBS) was prepared by mixing Na_2_HPO_4_ and NaH_2_PO_4_.

ITO-coated glasses (<17 Ω/square, thickness: 100 ± 20 nm) were purchased from Zhuhai Kaivo Optoelectronic Technology Co., Ltd. (China). To get a clean surface, the ITO electrode was immersed in 1 M NaOH solution overnight and then successively sonicated in acetone, ethanol, and ultrapure water. Ultrapure water (18.2 MΩ cm) used in the experiments was prepared by using the Milli-Q system (Millipore Company).

### 2.2 Measurements and instrumentations

Transmission electron microscopy (TEM) images were captured using a transmission electron microscope (JEM-2100, JEOL, Japan). Field-emission scanning electron microscopy (SEM) images and energy dispersive X-ray mapping spectroscopy (EDS mapping) data were analyzed using a scanning electron microscope (Sigma500, Zeiss, Germany). The X-ray photoelectron spectroscopy (XPS) data were collected on a PHI5300 electron spectrometer using 250 W, 14 kV, and Mg Kα radiation (PE Ltd., United States). All electrochemical measurements, including cyclic voltammetry (CV), electrochemical impedance spectroscopy (EIS), and differential pulse voltammetry (DPV), were conducted on a conventional three-compartment electrochemical cell by Autolab (PGSTAT302N) electrochemical workstation (Metrohm, Switzerland), with the modified ITO electrode, an Ag/AgCl electrode, and a platinum wire electrode as the working, reference, and counter electrodes, respectively. The scan rate for CV tests was 50 mV/s. The parameters for DPV measurements included step potential (0.005 V), pulse amplitude (0.05 V), pulse time (0.05 s), and interval time (0.2 s).

### 2.3 Preparation of SM@NH_2_-VMSF/ITO and GA/Pt NPs@NH_2_-VMSF/ITO electrodes

The NH_2_-VMSF/ITO could be grown on the bare ITO electrode (1 cm × 0.5 cm) by the electrochemically assisted self-assembly (EASA) method within 10 s (Etienne et al., 2009; Ma et al., 2022a; Ma N. et al., 2022). In brief, CTAB (1.585 g) was first dissolved in a mixture solution consisting of 0.1 M NaNO_3_ aqueous solution (20 mL, pH 2.6) and ethanol (20 mL). After the addition of APTES (0.318 mL), the pH of the mixed solution was adjusted to 2.97 using 6 M HCl. Subsequently, TEOS (2.372 mL) was added, and the obtained precursor solution was stirred at room temperature for 2.5 h. VMSF-bearing amino groups (NH_2_-VMSF) were prepared on the bare ITO electrode surface by immersing the clean ITO electrode in the aforementioned aged solution and applying a constant current density (−0.70 mA·cm^−2^) for 10 s. Then, the finally obtained electrode was rapidly washed with ultrapure water and aged for 12 h at 120°C. As for the directly as-prepared modified electrode, surfactant micelles (SMs) made up of CTAB were positioned inside the nanospace of silica nanochannels, designated as SM@NH_2_-VMSF/ITO.

GA-functionalized SM@NH_2_-VMSF/ITO, termed GA/SM@NH_2_-VMSF/ITO, was obtained using a simple drop-casting procedure, which could act as a cross-linking agent for further covalent immobilization of specifically recognized antibodies. Specifically, 5% GA (50 μL) was dropped onto the SM@NH_2_-VMSF/ITO electrode surface and incubated at 37°C for 30 min in a dark place. Then, the GA/SM@NH_2_-VMSF/ITO electrode was placed into a 0.1 M HCl/ethanol solution under stirring for 5 min to remove the SM, generating open channels for mass transport. Such resulting electrode was denoted as GA/NH_2_-VMSF/ITO. Pt nanoparticles were electrodeposited inside NH_2_-VMSF using chronoamperometry, and the growth of Pt NPs can be well controlled when applying a constant potential of −0.2 V for different durations. 3.86 mM of H_2_PtCl_6_·6H_2_O electrodeposition solution was composed of 100 mg/mL H_2_PtCl_6_·6H_2_O (1 mL) and 0.1 M H_2_SO_4_ (49 mL). In short, the GA/NH_2_-VMSF/ITO electrode was immersed in the aforementioned electrodeposition solution and applied a constant potential of −0.2 V for 2 s, finally achieving the Pt NPs confined into the nanochannels of NH_2_-VMSF, designated as GA/Pt NPs@NH_2_-VMSF/ITO.

### 2.4 Preparation of the electrochemical immunosensor based on the GA/Pt NPs@NH_2_-VMSF/ITO electrode and electrochemical determination of CEA

An electrochemical immunosensor for CEA detection was prepared by immersing the GA/Pt NPs@NH_2_-VMSF/ITO electrode in a 10 μg/mL antibody-CEA (50 μL) solution and incubating at 4°C for 1 h. After being rinsed with the residual antibody-CEA with 0.01 M PBS (pH 7.4), the immunosensing interface was eventually obtained, named the Ab/GA/Pt NPs@NH_2_-VMSF/ITO electrode.

CEA solution (50 μL) with various concentrations was dropped onto the Ab/GA/Pt NPs@NH_2_-VMSF/ITO electrochemical immunosensor and incubated at 4°C for 1 h. Then, the residual CEA solution was washed off using 0.01 M PBS (pH 7.4). DPV was utilized to measure the electrochemical signal of [Fe(CN)_6_]^3−^ before and after the interaction between Ab/GA/Pt NPs@NH_2_-VMSF/ITO and CEA. Moreover, the standard addition method was used for the determination of CEA in fetal bovine serum to prove the reliability of the constructed Ab/GA/Pt NPs@NH_2_-VMSF/ITO immunosensor in real samples. The fetal bovine serum was diluted by a factor of 50 using 0.01 M PBS (pH 7.4), and the CEA with a known concentration was added to the serum sample. Finally, the same detection procedure was conducted on the CEA detection in the serum sample using the Ab/GA/Pt NPs@NH_2_-VMSF/ITO electrochemical immunosensor.

## 3 Results and discussion

### 3.1 Preparation of a Pt NPs@NH_2_-VMSF/ITO-based immunosensor and its sensing mechanism for CEA

**SCHEME 1:**
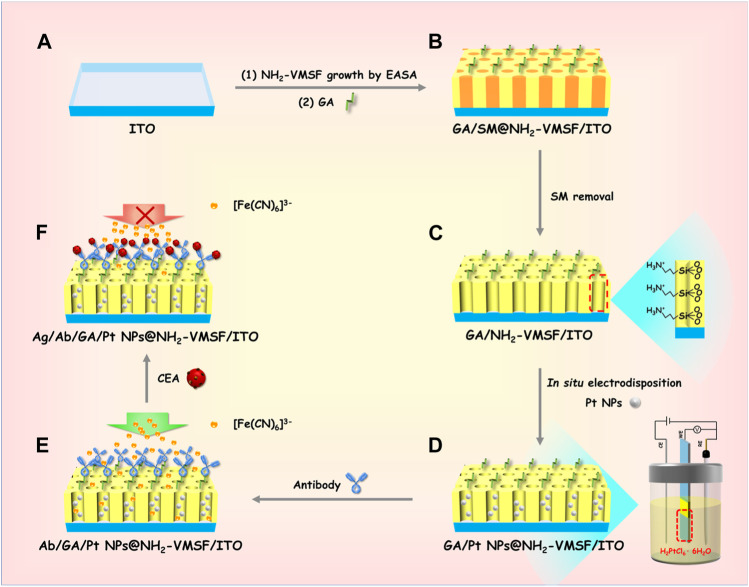
Schematic illustration of the preparation of the Ab/GA/Pt NPs@NH_2_-VMSF/ITO electrode **(A–E)** and its sensing mechanism for the CEA **(A–F)**.

Scheme 1 reveals the fabrication process of the ITO electrode decorated with NH_2_-VMSF, containing Pt nanostructures inside the inner nanochannels, while simultaneously modifying the anti-CEA antibody on the outmost surface using a convenient and controllable electrochemical method. The resulting electrode is termed as Ab/GA/Pt NPs@NH_2_-VMSF/ITO, combining the electrocatalyst effect of Pt NPs and the specific recognition capacity of the anti-CEA antibody. The growth of NH_2_-VMSF on the ITO electrode surface is accomplished by the EASA approach. Due to the presence of amine groups on both the inner silica walls and external surface, NH_2_-VMSF-encased surfactant micelles inside the nanochannels are used to functionalize with a linker agent (glutaraldehyde, GA) (denoted as GA/SM@NH_2_-VMSF/ITO, as shown in Scheme 1B), which can guarantee further modification of the anti-CEA antibody on the external surface of NH_2_-VMSF. After exclusion of SMs, NH_2_-VMSF possesses opened nanochannels and protonated amino groups on the silica walls, designated as GA/NH_2_-VMSF/ITO (Scheme 1C), which renders the active sites for electrosynthesis of Pt NPs *in situ* to obtain GA/Pt NPs@NH_2_-VMSF/ITO (Scheme 1D). Finally, the anti-CEA antibody is anchored to the external surface of NH_2_-VMSF through GA, achieving the Ab/GA/Pt NPs@NH_2_-VMSF/ITO sensor (Scheme 1E). The target CEA can be specially recognized on the Ab/GA/Pt NPs@NH_2_-VMSF/ITO sensing interface, resulting in the hampered mass transport of the [Fe(CN)_6_]^3–^ probe in the bulk solution to the underlying ITO electrode surface through the silica nanochannels of NH_2_-VMSF (Scheme 1F). Therefore, the decreased electrochemical current signal of [Fe(CN)_6_]^3–^ is associated with the CEA concentration, leading to the quantitative analytical method of detection of the CEA.

### 3.2 Characterization of NH_2_-VMSF/ITO and Pt NPs@NH_2_-VMSF/ITO electrodes

**FIGURE 1:**
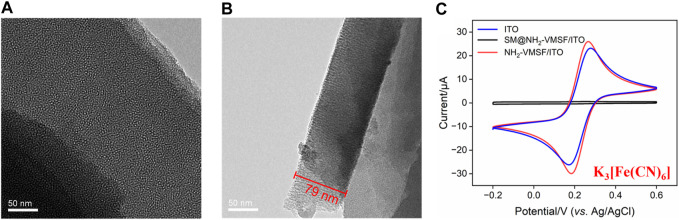
**(A)** Top-view TEM image of NH_2_-VMSF. **(B)** Cross-sectional TEM image of NH_2_-VMSF. **(C)** CV curves of the ITO, SM@NH_2_-VMSF/ITO, and NH_2_-VMSF/ITO electrodes in 0.05 M KHP containing 0.5 mM K_3_[Fe(CN)_6_].

Figure 1 depicts the transmission electron microscopy and electrochemical characterization of NH_2_-VMSF. A top-view TEM image of NH_2_-VMSF displays a crack-free structure with numerous uniform pores of nanometer-sized diameter (ca. 2–3 nm) (Figure 1A). As shown in Figure 1B, the nanochannel of the NH_2_-VMSF is homogeneous and its length is 79 nm. Figure 1C shows the CV curves of ITO, SM@NH_2_-VMSF/ITO, and NH_2_-VMSF/ITO electrodes in 0.05 M KHP containing 0.5 mM K_3_[Fe(CN)_6_]. The ITO electrode exhibits obvious redox peaks originating from the redox reaction of [Fe(CN)_6_]^3–^. However, the CV curve measured by the SM@NH_2_-VMSF/ITO electrode shows no obvious redox peak, which is attributed to the hydrophobic environment consisting of CTAB SM and leads to the obstructed transport of hydrophilic [Fe(CN)_6_]^3–^ within nanochannels. The NH_2_-VMSF/ITO electrode without SM inside the nanochannels not only has an open channel for free diffusion of [Fe(CN)_6_]^3–^ but also displays electrostatic attraction for negatively charged [Fe(CN)_6_]^3–^, eventually giving rise to amplified redox signals compared to those at the ITO. These results indicate that NH_2_-VMSF on the ITO electrode is intact without cracks, and the electrochemical response of [Fe(CN)_6_]^3–^ at the NH_2_-VMSF/ITO electrode can be enlarged, showing the significant potential of NH_2_-VMSF/ITO for the design of gated-controlled electrochemical sensors.

**FIGURE 2:**
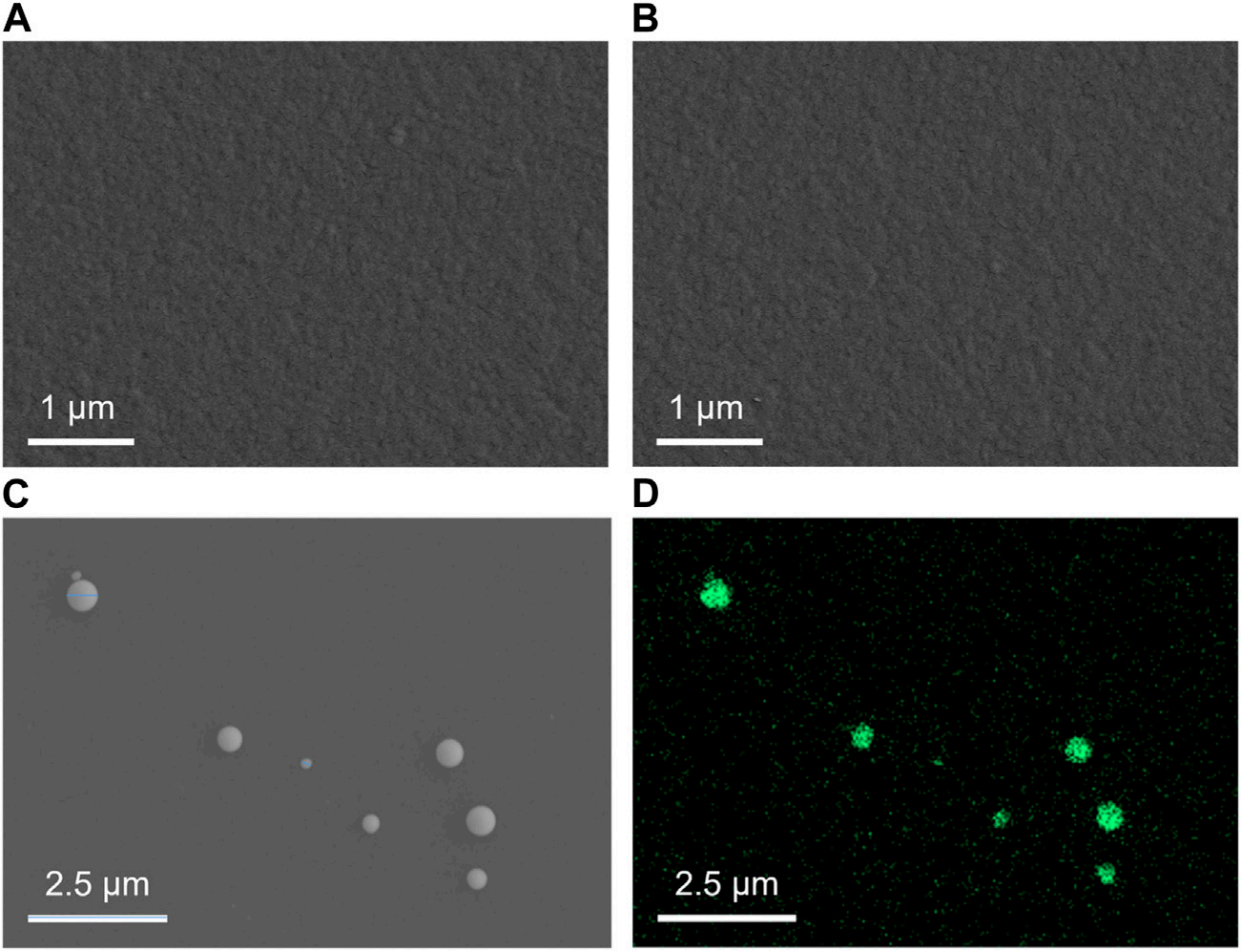
Top-view SEM images of NH_2_-VMSF/ITO **(A)** and Pt NPs@NH_2_-VMSF/ITO before **(B)** and after **(C)** the dissolution of NH_2_-VMSF. **(D)** EDS elemental mapping of Pt NPs at the Pt NPs@NH_2_-VMSF/ITO electrode after the dissolution of NH_2_-VMSF.

Top-view SEM images in Figures 2A, B show the surface of the NH_2_-VMSF/ITO electrode before and after the electrodeposition of Pt NPs. Both surfaces appear relatively smooth without obvious differences between NH_2_-VMSF/ITO and Pt NPs@NH_2_-VMSF/ITO, indicating that Pt NPs are confined inside the nanochannels. Figures 2C, D display the top-view SEM image and EDS elemental mapping of Pt NPs after dissolution of NH_2_-VMSF by 0.1 M NaOH (50 μL). As seen, Pt NPs distributed on the surface of the electrode have sizes ranging from 160 to 260 nm, which is probably due to the aggregation of Pt NPs after losing the protection of NH_2_-VMSF and also confirms the successful electrodeposition of Pt NPs within the nanochannels of NH_2_-VMSF.

**FIGURE 3:**
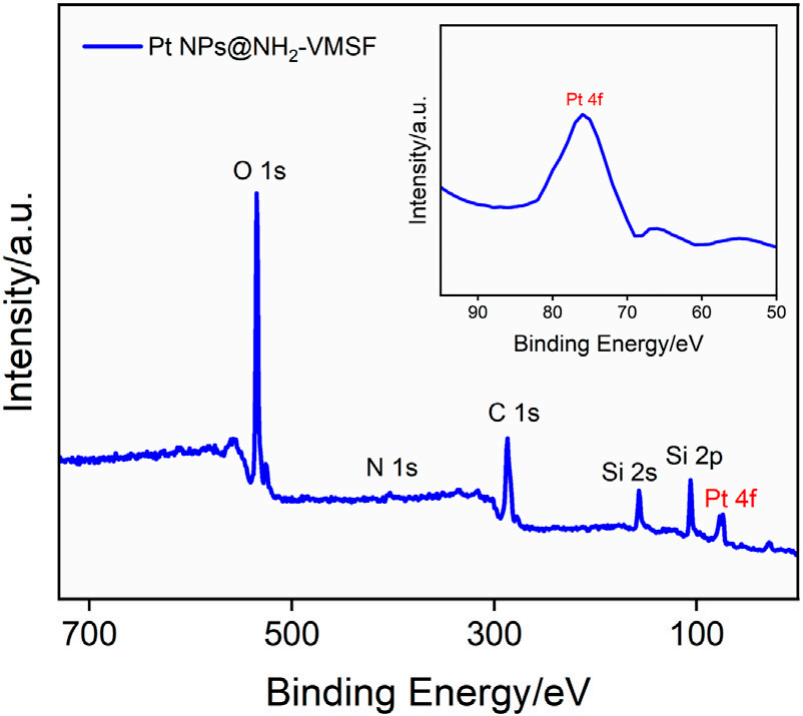
XPS survey spectra of Pt NPs@NH_2_-VMSF/ITO.

To further verify the successful electrodeposition of Pt NPs into the nanochannels of NH_2_-VMSF, XPS analysis was conducted, as shown in Figure 3. As demonstrated, the presence of N and Pt in XPS data is derived from amino groups of NH_2_-VMSF and electrodeposited Pt NPs, respectively. In addition, C, O, and Si elements are from the NH_2_-VMSF structure. All the aforementioned results confirm the successful preparation of the Pt NPs@NH_2_-VMSF/ITO electrode.

### 3.3 Characterization of the Pt NPs@NH_2_-VMSF/ITO-based electrochemical immunosensor

**FIGURE 4:**
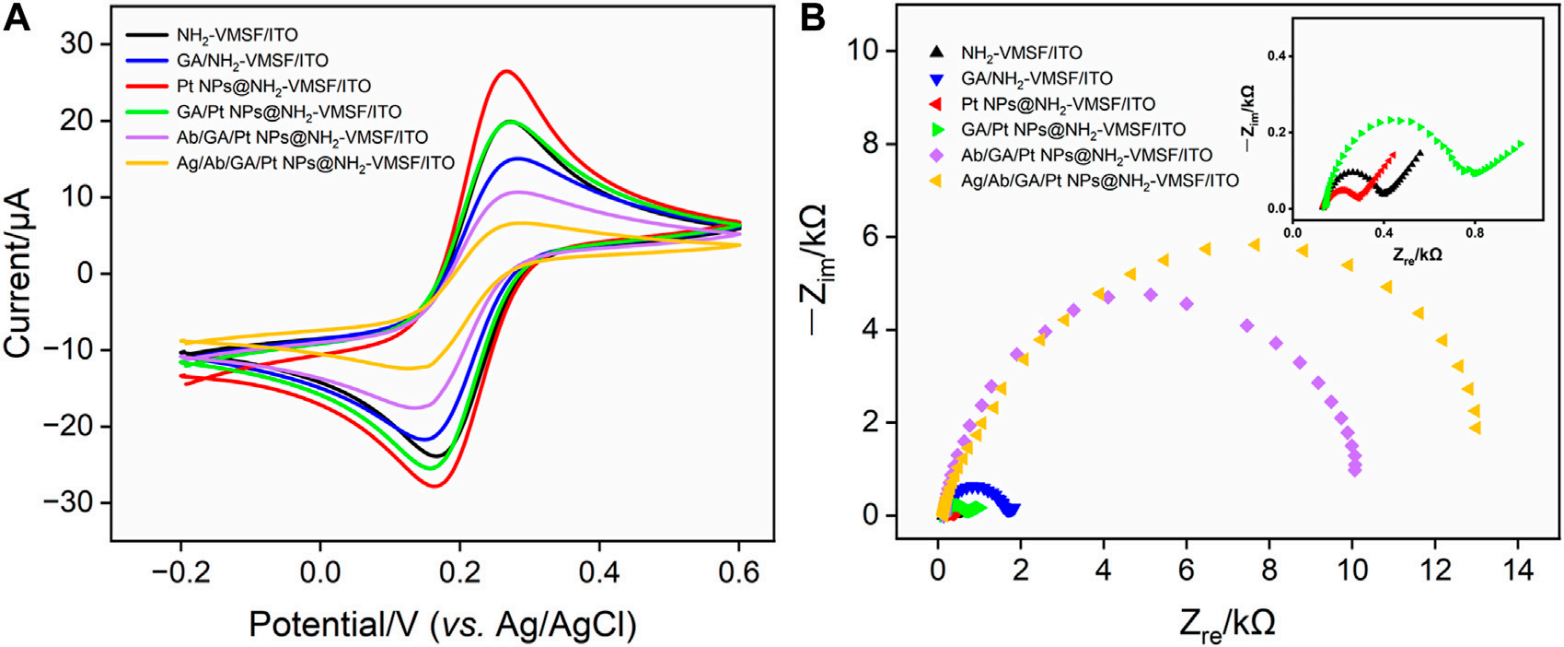
**(A)** CV curves of different electrodes in 50 mM KHP containing 0.5 mM K_3_[Fe(CN)_6_] and **(B)** EIS curves of different electrodes in 0.1 M KCl containing 2.5 mM [Fe(CN)_6_]^3–^/[Fe(CN)_6_]^4–^.

CV and EIS were used as electrochemical methods to investigate the interfacial state changes during the construction of the Ab/GA/Pt NPs@NH_2_-VMSF/ITO sensor. Figure 4 displays the CV (A) and EIS (B) curves of NH_2_-VMSF/ITO, GA/NH_2_-VMSF/ITO, Pt NPs@NH_2_-VMSF/ITO, GA/Pt NPs@NH_2_-VMSF/ITO, Ab/GA/Pt NPs@NH_2_-VMSF/ITO, and Ag/Ab/GA/Pt NPs@NH_2_-VMSF/ITO electrodes in 50 mM KHP containing 0.5 mM [Fe(CN)_6_]^3–^. The cross-linking of GA with amino groups at the entrance of the silica nanochannels causes a decrease in redox peak current values of [Fe(CN)_6_]^3–^ at the GA/NH_2_-VMSF/ITO and GA/Pt NPs@NH_2_-VMSF/ITO, in comparison with those obtained at the NH_2_-VMSF/ITO and Pt NPs@NH_2_-VMSF/ITO (Figure 4A). After the successful electrodeposition of Pt NPs into the nanochannels of NH_2_-VMSF, the magnitude of redox peak currents significantly increased due to the signal amplification capacity of Pt NPs. The redox peak currents further decrease after covalently attaching Ab to the GA/Pt NPs@NH_2_-VMSF/ITO surface, which is attributed to the insulating property of proteins hindering the mass transfer of electrons on the electrode surface. Upon immobilization of CEA, the redox current values remarkably decrease, confirming the successful formation of the antibody–antigen immunocomplex at the sensing interface. EIS plots shown in Figure 4B consist of a semicircle part in the high-frequency region and a linear part in the low-frequency region, which are associated with the electron transfer and diffusion processes, respectively. Electron transfer resistance (*R*_ct_) can be extracted from the magnitude of the semicircle diameter, showing the electron transfer variation between different electrodes. EIS measurements in Figure 4B reveal *R*_ct_ of the same electrodes shown in Figure 4A. Similar variation is observed, and GA/Pt NPs@NH_2_-VMSF/ITO exhibits lower *R*_ct_ compared to GA/NH_2_-VMSF/ITO, indicating that Pt NPs, as a kind of excellent electronic conductivity material, improve electron transfer ability on the electrode interface. Moreover, *R*_ct_ significantly increases after immobilization of the anti-CEA antibody and target CEA, demonstrating that the formed antibody–antigen immunocomplex indeed obstructs the diffusion of electrons and further proving the feasible detection capacity of the developed Ab/GA/Pt NPs@NH_2_-VMSF/ITO.

### 3.4 Optimization of experimental conditions

**FIGURE 5:**
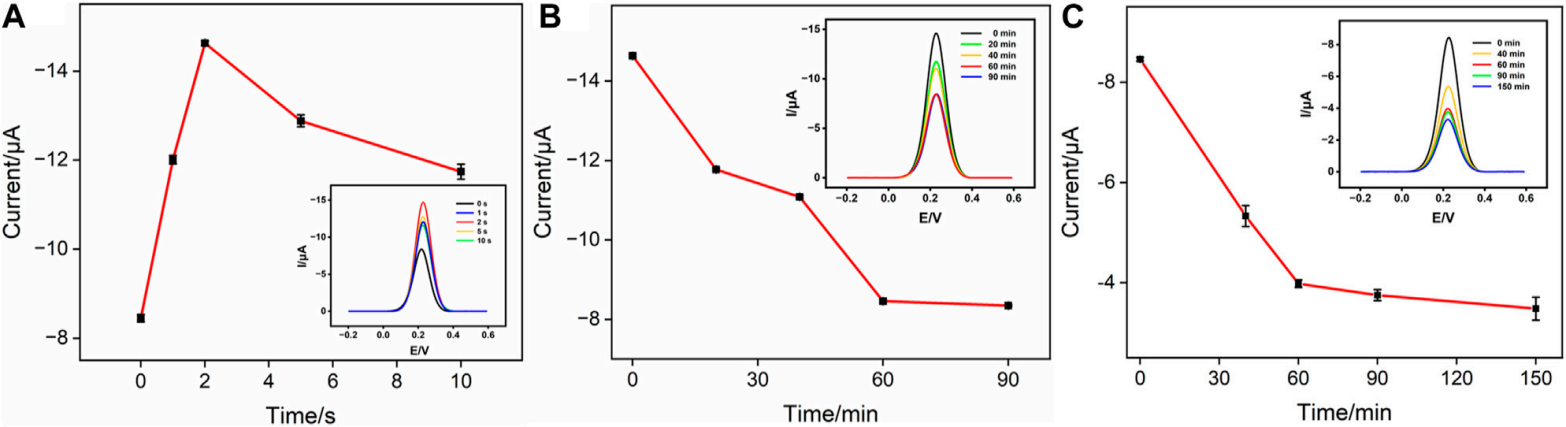
**(A)** Cathodic peak currents measured at the GA/Pt NPs@NH_2_-VMSF/ITO in 50 mM KHP containing 0.5 mM K_3_[Fe(CN)_6_] at different deposition times of Pt NPs. The inset shows its corresponding DPV curves. **(B)** Cathodic peak currents measured at the Ab/GA/Pt NPs@NH_2_-VMSF/ITO in 50 mM KHP containing 0.5 mM K_3_[Fe(CN)_6_] at different incubation times for the anti-CEA antibody. The inset shows its corresponding DPV curves. **(C)** Cathodic peak currents measured at the Ag/Ab/GA/Pt NPs@NH_2_-VMSF/ITO in 50 mM KHP containing 0.5 mM K_3_[Fe(CN)_6_] at different incubation times for the CEA. The inset shows its corresponding DPV curves.

There are several factors that affect the analytical performance of the Ab/GA/Pt NPs@NH_2_-VMSF/ITO sensor, including the electrodeposition time of Pt NPs and the incubation time of the anti-CEA antibody or CEA. First, we studied the performance of the developed sensor with various electrodeposition times of Pt NPs ranging from 1 s to 10 s (Figure 5A). When the electrodeposition time increases from 1 s to 2 s, the obtained DPV signal for CEA increases due to the increased amount of Pt NPs and reaches its maximum when the deposition time is set to 2 s. When the electrodeposition time further increases from 2 s to 10 s, the signal gradually decreases. This decrease can be attributed to the reduced effective space of NH_2_-VMSF’s nanochannels for accessible transport of the [Fe(CN)_6_]^3–^ probe in the bulk solution. Subsequently, we investigated the incubation time of the anti-CEA antibody. As displayed in Figure 5B, it could be found that the peak current intensity decreases as the incubation time increases up to 60 min. This is because the amount of anti-CEA immobilized on the electrode is approaching saturation. Similarly, the effect of incubation time for the CEA on the electrochemical response is shown in Figure 5C. It is evident that as the incubation time increases, the peak current signal gradually decreases. After incubation for more than 60 min, the signal changes become minimal, indicating that the immunocomplex formed between the CEA and anti-CEA antibody has reached saturation. Therefore, 60 min is determined to be the optimal incubation time.

### 3.5 Quantitative determination of CEA using the fabricated Ab/GA/Pt NPs@NH_2_-VMSF/ITO immunosensor

**FIGURE 6:**
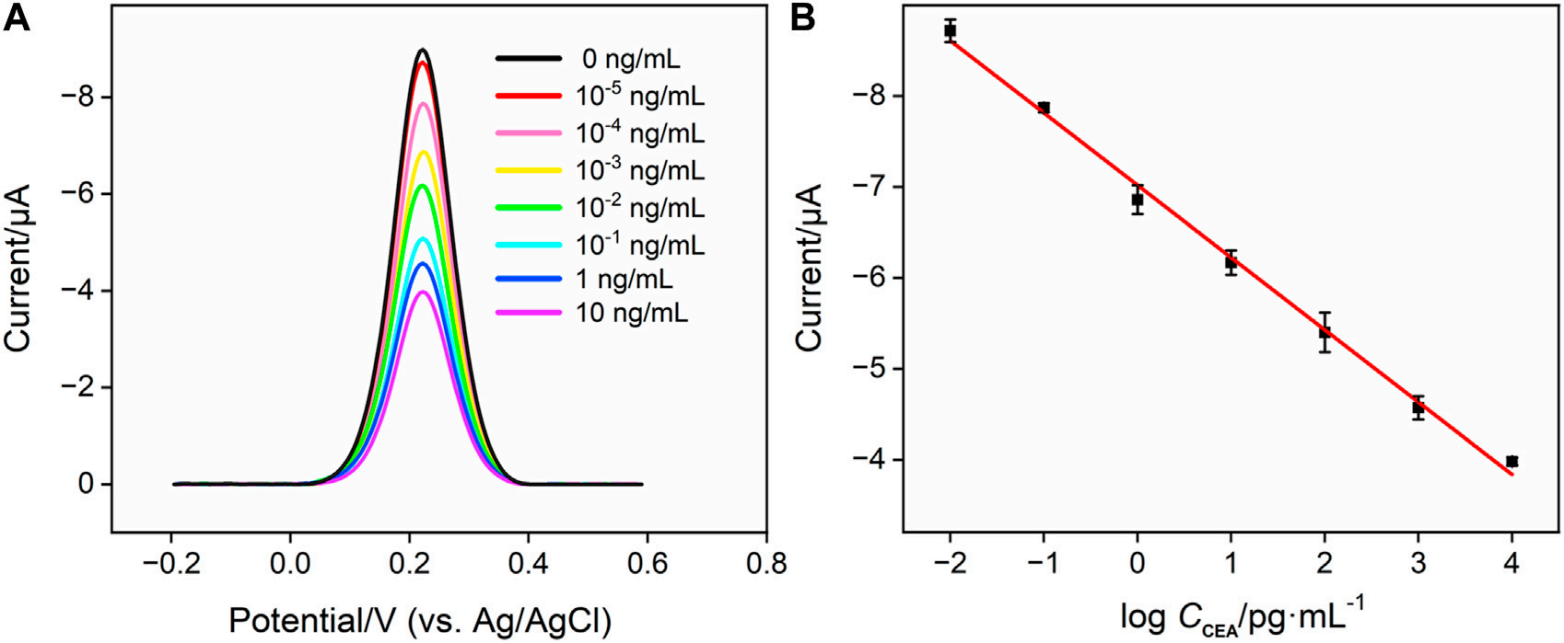
**(A)** DPV responses of the Ag/Ab/GA/Pt NPs@NH_2_-VMSF electrode to various concentrations of CEA in 0.01 M PBS (pH 7.4). **(B)** Corresponding calibration curves. Error bars refer to the standard deviations of three measurements.

To evaluate the analytical performance of the Ab/GA/Pt NPs@NH_2_-VMSF/ITO sensor, we tested it in detecting the CEA with various concentrations under optimized conditions using the DPV technique. As shown in Figure 6A, the cathodic peak current decreased progressively with the increase in the concentration of the CEA due to the continuous formation of the antibody–antigen complex at the sensing interface. A good linear relationship is displayed between the DPV signal (*I*_DPV_) and the logarithm of the CEA concentration (*C*_CEA_) in the range of 0.01 pg/mL to 10 ng/mL (Figure 6B), yielding a linear regression equation of *I*_DPV_ (μA) = 0.796 log*C*_CEA_-7.02 (*R* ^2^ = 0.996). Furthermore, the limit of detection (LOD) calculated is 0.3 fg/mL at the signal-to-noise ratio of 3 (S/N = 3). We compare the related analytical parameters and construction strategy of the Ab/GA/Pt NPs@NH_2_-VMSF/ITO sensor with the other reported sensors. As shown in Table 1, our fabricated Ab/GA/Pt NPs@NH_2_-VMSF/ITO strategy has a low LOD and simple preparation steps. Moreover, the developed sensor not only achieves dual signal amplification through the electrocatalysis ability of Pt NPs and the electrostatic enrichment effect of VMSF at the electrode interface but also has the advantage of a shorter construction time.

**TABLE 1:** Comparison of the analytical performances of different methods for the determination of the CEA.

Sensing platform	Method	Detection range (ng/mL)	LOD (pg/mL)	Step	Construction time (h)	Reference
BSA/Ab/Pd@Pt/MoS_2_-Gr/GCE	EC	0.00001–100	0.005	13	39	Lin et al. (2021)
CuO NPs/Ag/BSA/Ab/MB	Colorimetry	0.05–100	26	8	5	Li et al. (2017)
PdCu-Ab_2_/Ag/Ab_1_/BSA/AuNPs/GCE	EC	0.0001–10	0.08	7	16	Jiao et al. (2017)
GO-PEI-Ru-AuNPs-Ab_2_/Ag/Ab_1_/AuNFs/pL-Cys/GCE	ECL	0.0001–80	0.045	12	37	Yuan et al. (2018)
CEA aptamer/ZnS-CdS/MoS_2_/GCE	ECL	0.05–20	30	8	60	Wang et al. (2016)
Ab/AuNPs@nafion/FC@CHIT/GCE	EC	0.03–100	10	9	8	Shi and Ma (2011)
Ab/GA/Pt NPs@NH_2_-VMSF/ITO	EC	0.0001–10	0.0003	7	5	This work

BSA, bovine serum albumin; Ab, the antibody of the CEA; MoS_2_, molybdenum disulfide; Gr, graphene; GCE, glassy carbon electrode; CuO NPs, copper oxide nanoparticle; Ag, carcinoembryonic antigen; MB, magnetic bead; PdCu, porous PdCu nanoparticles; AuNPs, gold nanoparticles; GO, graphene oxide; PEI, polyethylenimine; Ru, the luminophor tris (4,40-dicarboxylicacid-2, 20-bipyridyl) ruthenium (II) dichloride (Ru(dcbpy)_3_^2+^); AuNFs, flower-like gold nanoparticles; pL-Cys, polyamino acid L-cysteine; ZnS–CdS, ZnS–CdS nanoparticle; FC: K_3_[Fe(CN)_6_]; CHIT, chitosan.

### 3.6 Selectivity of the fabricated Ab/GA/Pt NPs@NH_2_-VMSF/ITO immunosensor

**FIGURE 7:**
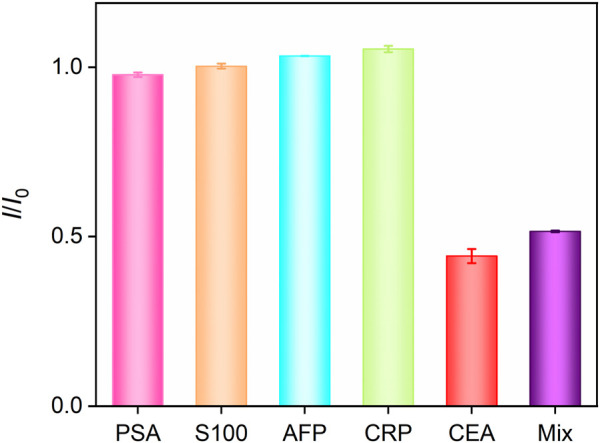
DPV current ratio obtained from Ab/GA/Pt NPs@NH_2_-VMSF/ITO in the absence (*I*_*0*_) or presence (*I*) of different species with the same concentration (10 ng/mL). Error bars refer to the standard deviations of three measurements.

The selectivity of the fabricated Ab/GA/Pt NPs@NH_2_-VMSF/ITO sensor were studied by multiple potential interfering substances, including the PSA, S100 calcium-binding protein *β*, AFP, and CRP. As shown in Figure 7, the Ab/GA/Pt NPs@NH_2_-VMSF/ITO immunosensor demonstrates excellent signal response to the CEA and a mixture of the aforementioned substances containing the CEA while showing almost no response to other interfering substances. This indicates that our sensing platform has high selectivity to the CEA, resulting from the specific binding of the anti-CEA antibody and CEA complex.

### 3.7 Detection of CEA in real samples

To validate the reliability and accuracy of the developed Ab/GA/Pt NPs@NH_2_-VMSF/ITO sensor, we conducted real sample testing by detecting the CEA amount in fetal bovine serum using the standard addition method. After diluting the fetal bovine serum by a factor of 50 using 0.01 M PBS (pH 7.4), we added 0.1 pg/mL, 10 pg/mL, and 1000 pg/mL of CEA and measured the electrochemical signals by DPV. The added known and tested concentrations of the CEA are designated as “*C*_added_” and “*C*_found_”. In addition, the recovery is defined as the concentration ratio ((*C*_added_/*C*_found_)×100%), which is used to evaluate the detection performance of the fabricated Ab/GA/Pt NPs@NH_2_-VMSF/ITO in real samples. Generally, recovery ranging from 90.0% to 110% is considered for high accuracy. The relative standard deviation (RSD) value represents the deviation of three measurements disseminated around the average value, which is expressed as the ratio of the standard deviation to the average value. The lower the RSD, the closer the measured values are to the average value, indicating good precision. As shown in Table 2, the recoveries of the CEA obtained in the aforementioned fetal bovine serum samples range from 104.5% to 107.4% with low RSD values (<5.7%). These results demonstrate the promising potential of the proposed Ab/GA/Pt NPs@NH_2_-VMSF/ITO sensor for sensitive detection of the CEA in clinical applications.

**TABLE 2:** Recoveries of the CEA in the fetal bovine serum sample (*n* = 3).

Sample	*C*_Added_ (pg·mL^–1^)	*C*_Found_ (pg·mL^–1^)	Recovery (%)	RSD (%)
Serum	0.1000	0.1060	106.0	3.1
	10.00	10.45	104.5	5.7
	1000	1074	107.4	2.8

## 4 Conclusion

In summary, Pt NPs confined in the silica nanochannels of NH_2_-VMSF without any protecting ligands have been successfully synthesized on the ITO electrode surface using a simple electrochemical method. The CEA antibody covalently modified on the external surface of NH_2_-VMSF endows the sensor with good specificity for CEA detection. With the help of the [Fe(CN)_6_]^3−^ probe in the bulk solution, the high affinity between the CEA antibody and CEA on the sensing interface forms the steric hindrance effect for the accessibility of [Fe(CN)_6_]^3−^ to the underlying ITO surface, resulting in an attenuated electrochemical signal and allowing the detection of the CEA with a wide linear range of 0.01 pg/mL∼10 ng/mL and a pretty low limit of detection of 0.30 fg/mL. Combining the signal amplification ability of Pt NPs and the anti-biofouling property of NH_2_-VMSF, the presented sensing strategy can be directly applied in detecting the CEA in human serum samples, which is helpful for the analysis of tumor-related biomarkers in clinical diagnosis.

## Data Availability

The original contributions presented in the study are included in the article/Supplementary material; further inquiries can be directed to the corresponding authors.
